# Autoimmune glial fibrillary acidic protein astrocytopathy complicated with low flow perimedullary arteriovenous fistula: a case report

**DOI:** 10.3389/fimmu.2023.1293425

**Published:** 2023-12-04

**Authors:** Ting Xu, Jingyun Chen, Tingting Xuan, Jiang Cheng, Haining Li

**Affiliations:** ^1^Department of Neuroelectrophysiology, Cardiovascular and Cerebrovascular Disease Hospital Branch, General Hospital of Ningxia Medical University, Yinchuan, China; ^2^School of Clinical Medicine, Ningxia Medical University, Yinchuan, China; ^3^Diagnosis and Treatment Engineering Technology Research Center of Nevous System Disease of Ningxia Hui Autonomous Region, Yinchuan, China; ^4^Department of Neurology, Cardiovascular and Cerebrovascular Disease Hospital Branch, General Hospital of Ningxia Medical University, Yinchuan, China; ^5^Department of Neurology, General Hospital of Ningxia Medical University, Yinchuan, China

**Keywords:** autoimmune disease, glial fiber acidic protein, spinal vascular malformation, low flow, perimedullary arteriovenous fistula

## Abstract

Autoimmune glial fibrillary acidic protein (GFAP) astrocytopathy and low-flow perimedullary arteriovenous fistulas (PMAVFs) may cause longitudinal widespread myelopathy. We report a middle-aged male patient with autoimmune GFAP astrocytopathy complicated with low flow PMAVFs disease, presenting with lower extremity weakness and dysuria. Magnetic resonance imaging (MRI) of the spinal cord revealed a significant longitudinal extent of T2 high signal from T11 to L1, with the lesion located proximal to the vascular territory supplied by the anterior spinal artery. Multiple patchy abnormal signals were seen adjacent to the anterior and posterior horns of the lateral ventricles bilaterally and at the centers of the semi-ovals on MRI of the cranial brain, with iso signal in T1Flair, the high signal in T2WI, and no high signal seen in Diffusion Weighted Imaging (DWI). Subsequently, the presence of anti-GFAP antibodies was detected in the cerebrospinal fluid (CSF), and the diagnosis of autoimmune GFAP astrocytopathy in conjunction with low-flow PMAVFs was confirmed through spinal digital subtraction angiography (DSA). This case report aims to increase neurologists’ awareness of this disease and avoid missed or misdiagnosed cases that may lead to delayed treatment.

## Introduction

Autoimmune GFAP astrocytopathy is an autoimmune-mediated disease of the central nervous system (CNS) that can affect the entire neuraxis and was first proposed in 2016 (1). Most of the disease has flu-like prodromal infection symptoms, which manifest as nonspecific subacute meningeal symptoms (headache, fever, photophobia, neck stiffness), myelopathy symptoms (weakness or numbness), memory loss, blurred consciousness, or blurred vision (optic disc edema), and other symptoms including dyskinesia, autonomic dysfunction, and cerebellar ataxia (1–3). Cerebrospinal fluid (CSF) in autoimmune GFAP astrocytopathy usually shows an inflammatory pattern with a predominance of lymphocytes, elevated protein levels, and elevated immunoglobulins (2, 4, 5). Typical imaging manifestations are abnormal T2 signaling in the periventricular white matter on cranial MRI as well as perivascular enhancement of line-like radiolucencies perpendicular to the lateral ventricles following the administration of gadolinium-containing contrast agents and longitudinally extensive T2 hyperintensities on spinal MRI. GFAP antibodies in the cerebrospinal fluid are its specific biomarker (2, 4) and most patients respond to steroids, but some cases are refractory and require second-line therapy (2, 5, 6). Early diagnosis of this inflammatory CNS disease remains challenging.

Spinal vascular malformations account for approximately 5%-9% of vascular malformations in the central nervous system (7). Low-flow spinal arteriovenous fistulas are more common, accounting for 80% of spinal arteriovenous malformations. Low-flow PMAVFs are one of the low-flow spinal arteriovenous fistulas, which are acquired lesions usually secondary to venous hypertension of the spinal cord in elderly patients with progressive spinal cord lesions (8). Low-flow PMAVFs are slow shunts between the anterior spinal arteries and veins that usually occur on the surface of the spinal cord or at the anterior median fissure of the spinal cord (9, 10). In 86% of patients with low-flow arteriovenous fistulae, spinal cord MRI reveals longitudinally extensive intramedullary T2 high signal (11). Due to its lack of specificity in clinical presentation and imaging, it frequently exhibits similarities to inflammatory myelopathy, leading to frequent instances of oversight or misdiagnosis. Because its clinical presentation and imaging are nonspecific, it can often mimic inflammatory myelopathy and is easily missed or misdiagnosed. Despite increased awareness of this condition and advances in surgical skills in recent years, the diagnosis of perimedullary arteriovenous fistulae involving low-flow fistulas remains a challenge. While other spinal vascular malformations have high-flow patterns that are easily recognized by MRI, low-flow vascular malformations are often missed or misdiagnosed due to their diverse clinical presentations and lack of specificity on imaging (12). Digital subtraction spinal angiography is its gold standard, and steroid treatment is associated with significant worsening of the condition (12–14). Early recognition and surgical intervention can lead to a favorable prognosis.

Autoimmune GFAP astrocytopathy and PMAVFs are two rare disorders, both of which can cause longitudinally extensive myelopathy (1, 4, 11). There is no known association between autoimmune GFAP astrocytopathy and PMAVFs, and there is no record of their coexistence. Here we present a case of a male patient case with longitudinal extensive myelopathy that was coexisting with PMAVFs in the context of autoimmune GFAP astrocytopathy. We discuss the similarities and differences between the two with the aim of enabling neurologists to better recognize such diseases, make an early diagnosis, and avoid misdiagnosis and missed diagnoses leading to treatment delays.

## Case report

A 49-year-old male patient showed left lower limb weakness for 8 days with urinary retention for 6 days, the local hospital lumbar spine MRI showed that from L3 to S1 disc degeneration and backward mild bulging, considering the possible lumbar disc herniation due to compression of the nerve, the patient’s limb weakness was relieved by the treatment of hepcidium saponin sodium and dexamethasone, but he still felt difficulty in urination, and in order to further clarify the cause of the disease, he was admitted to the emergency room of our hospital. After admission, his body temperature was 36.6°C, pulse rate was 79 beats/min, blood pressure was 125/85 mmHg. Neurological examination showed that the muscle strength of the left lower limb was grade 4+, Babinski’s sign was positive bilaterally, and the left tic reflex disappeared. Other systemic examinations showed no abnormalities. He denied a history of hot flashes, night sweats, arthritis, cough, shortness of breath, diarrhea, and back problems. He denied any personal history of mycobacterium tuberculosis infection or contact with cattle or sheep. The patient was admitted to the neurology ward with “spinal cord lesions of a nature to be determined”.

**Figure 1:**
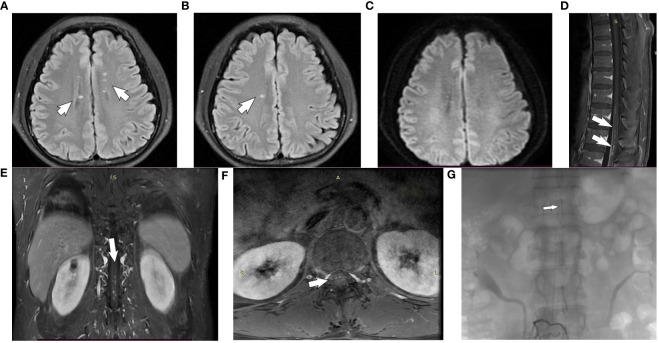
Brain and spinal MRI of a subject with autoimmune GFAP astrocytopathy. **(A, B)** The cranial MRI T2 FLAIR sequences: multiple patchy abnormal signals beside the anterior and posterior horns of the bilateral lateral ventricles and in the center of the semiovals(white arrow). **(C)** Diffusion Weighted Imaging(no high signal seen). **(D–F)** The gadolinium-enhanced T1-weighted image shows patchy enhancement of thoracic spine 11- lumbar spine 1 (white arrows). **(G)** Spinal digital subtraction angiography: the third lumbar artery on the right side of the vertebral canal to the supply of the anterior spinal artery in the spinal canal about the thoracic 10 position(white arrows).

Cranial MRI showed multiple patchy abnormal signals beside the anterior and posterior horns of the bilateral lateral ventricles and in the center of the semiovals, with isosignal in T1Flair, high signal in T2WI, and no high signal in DWI (**Figures 1A–C**). MRI/Magnetic Resonance Angiography (MRA) of thoracolumbar segment showed striated long T2 signal foci from T11-L1 spinal cord, enhancement was visible; striated T2WI sequence slightly high signal shadow was seen in the spinal cord at the level of T11-12 vertebral body, enhancement scan was visible as striated enhancement, and vascular malformation was considered (**Figures 1D–F**). Electromyography showed left posterior tibial nerve H-reflex with normal latency and decreased wave amplitude.

**Figure 2:**
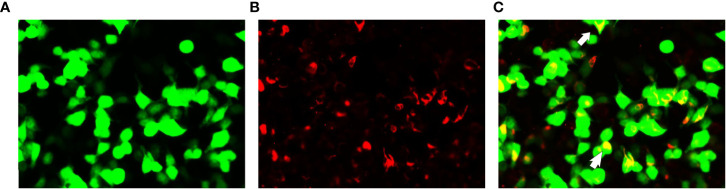
Glial fibrillary acidic protein (GFAP-IgG) by GFAP-transfected cell-based immunofluorescence assay. Cells were expressing green fluorescent protein-tagged GFAP (green) and immunostained (red if positive). **(A–C)** Examination of cerebrospinal fluid (CSF) in first admission. Merged images revealed the colocalization of the GFAP antibody and astrocyte (white arrows).

Lumbar puncture revealed a CSF pressure of 150 mmH_2_O, a leukocyte count of 5/mm^3^, and 0 lymphocytes, monocytes, and centrocytes; elevated protein of 0.98 g/L (0.12-0.60); normal glucose of 2.8 mmol/L; and elevated cerebrospinal fluid immunoglobulin G of 70 mg/L (0-34) (**Table 1**). Serologic testing was performed, including infectious, rheumatologic, and neurologic etiologies, and this testing was unremarkable. Serum/CSF was positive for anti-GFAP antibodies (1:32) (**Figures 2A–C**) and negative for anti-AQP4, anti-MOG and anti-NMDAR. The diagnosis of autoimmune GFAP astrocytopathy was clear in combination with the patient’s cranial MRI and spinal MRI.

**Table 1:** Changes in cerebrospinal fluid parameters during disease.

	Day13
CSF pressure(mmH_2_O)(80-180)	150
White cell count (10^6^/L)(0-10)	5 (0% lymphocyte/0% neutrophil)
Red cell count (10^6^/L)	0
Total protein (g/L)(0.12-0.60)	0.98
CSF glucose (mmol/L)(2.2-3.9)	2.8
CSF immunoglobulin G(mg/L)(0-34)	70

*CSF, cerebrospinal fluid.

Neurosurgery interventional group, radiology neurologist read the spinal MRI/MRA, considering that the patient’s lesion is located in the ventral side near the anterior spinal artery blood supply region, can not be excluded from the combination of spinal vascular malformation (spinal arteriovenous fistula), because arteriovenous fistula after the use of steroids will further deteriorate the condition, need to carry out a DSA, confirmed that the third lumbar artery on the right side of the vertebral canal to the supply of the anterior spinal artery in the spinal canal about the thoracic 10 position (**Figure 1G**). Due to the very small fistulae of PMAVFs, as well as the improvement of the patient’s symptoms with out-of-hospital dexamethasone, after a comprehensive evaluation, the patient was considered to have this symptom due to autoimmune GFAP astrocytopathy, and was given methylprednisolone 500 mg (×3d) intravenously, which was changed to 250 mg (×3d) after 3 days, and then changed to prednisone acetate 60 mg/d orally after 3 days.

After steroid treatment the patient’s left lower limb numbness and weakness gradually improved, she could walk on the ground, and her urinary function gradually recovered. In view of the association between GFAP astrocytopathy and malignant neoplasms, the patient underwent relevant investigations such as chest CT, abdominal ultrasound, urinary ultrasound, and tumor markers, and no evidence of occult malignancy was found. Oral hormone therapy was continued out of hospital. At the time of writing (13 months after discharge), he has no recurrent neurologic deficits.

## Discussion and conclusion

This case describes a case of autoimmune GFAP astrocytopathy combined with a low-flow PMAVFs that was shown to be extremely small on spinal angiography. Together with the patient’s symptomatic improvement after out-of-hospital dexamethasone use and subsequent high-dose steroid therapy, a comprehensive evaluation of the patient’s presenting clinical symptoms considered that this clinical condition was primarily due to autoimmune GFAP astrocytopathy. Despite the absence of significant clinical symptoms in this particular case, the presence of low-flow PMAVFs often poses a diagnostic challenge for clinicians due to its resemblance in clinical manifestations and radiologic characteristics to autoimmune GFAP astrocytopathy.The neurologic sequelae can be devastating if left untreated or empirically given steroids.

Myelitis is a common feature of autoimmune GFAP astrocytopathy. Among 19 patients with autoimmune GFAP astrocytopathy included by Long et al, 16 patients underwent spinal cord MRI, and 11 patients (81.25%) had longitudinally extensive myelopathy, with all cases showing central spinal gray matter involvement (4). The first symptom of the patient in this paper was lower limb weakness with spinal cord involvement, and autonomic dysfunction (dysuria) gradually appeared with the progression of the disease, and perfect MRI of the thoracic and lumbar vertebrae showed intramedullary T2 high-signal lesions extending from T11 to the L1 vertebral segment, which was consistent with the previous reports in the literature (1–3). As the spinal cord lesion in this patient was biased to the ventral region of the anterior spinal artery supply, limb weakness and dysuria due to vascular myelopathy (spinal arteriovenous fistula) could not be excluded. In atypical clinical situations, vascular myelopathy can easily be overlooked and diagnosed as other neurological disorders such as demyelinating diseases, including acute transverse myelitis, multiple sclerosis, or optic neuromyelitis optica (15). This is a difficulty in pre-diagnosis.

Lower extremity weakness and dysuria, as well as longitudinal widespread T2 high signal on spinal MRI, have been reported in patients with low-flow PMAVFs and are easily misdiagnosed as inflammatory myelitis (12, 16). Although there are similarities between the two, there are some clinically important clues to identify these two diseases in this article. In this case, the patient’s cranial MRI showed multiple patchy abnormal signals seen adjacent to the anterior and posterior horns of the bilateral lateral ventricles and in the center of the semiovals, with isosignal T1Flair, high signal T2WI, and inflammatory manifestations with no high signal on DWI, which was in agreement with the reports related to the preexisting autoimmune GFAP astrocytopathy (2, 4). This is not visible in low-flow PMAVFs. It is important for us to note that when a patient’s clinical symptoms consider autoimmune GFAP astrocytopathy, early refinement of cranial MRI is required, even in the absence of symptoms involving the meninges and brain parenchyma.

CSF in autoimmune GFAP astrocytopathy usually shows an inflammatory pattern manifested by increased lymphocyte counts and elevated proteins (2, 4). However, in this paper, the patient’s CSF had a normal leukocyte count and showed only elevated protein and immunoglobulin, which was also inconsistent with previous literature reports, and was considered to be possibly related to the patient’s out-of-hospital dexamethasone use. Although low-flow PMAVFs can also present with elevated cerebrospinal fluid cell counts and proteins, the cerebrospinal fluid usually has a leukocyte count of <12 cells/mm^3^ and mildly elevated proteins (12). This is one of the points of differentiation between the two.

The use of steroids improves clinical symptoms of autoimmune GFAP astrocytopathy (1, 2), but causes rapid deterioration of clinical symptoms in patients with perimedullary arteriovenous fistulae (12–14). In this case, the patient’s limb weakness symptoms improved after administration of dexamethasone treatment given locally prior to hospitalization, and the symptoms improved significantly after subsequent high-dose steroid shock therapy, further suggesting that the patient’s clinical symptoms on this occasion were related to autoimmune GFAP astrocytopathy, which is one of the points of differentiation between the two. Of course, GFAP IgG positivity in CSF is the basis for the final diagnosis of autoimmune GFAP astrocytopathy, while spinal angiography is the basis for the diagnosis of perimedullary arteriovenous fistula. The asymptomatic consideration of PMAVFs in this case may be (1) The fistula is extremely small and the fractional flow is minimal. (2) Because the spinal radicular veins occlude with age due to fibrosis, excessive blood flow through a low-flow PMAVFs in the early stages does not cause neurological deficits (17).

In conclusion, autoimmune glial fibrillary acidic protein astrocytopathy and low-flow spinal arteriovenous fistulae overlap in clinical presentation and spinal MRI, and timely spinal angiography and detection of GFP antibodies are crucial for patient prognosis.

## Data availability statement

The original contributions presented in the study are included in the article/supplementary material. Further inquiries can be directed to the corresponding authors.

## Ethics statement

The studies involving humans were approved by Member of Medical Research Ethics Review Committee, General Hospital of Ningxia Medical University. The studies were conducted in accordance with the local legislation and institutional requirements. The participants provided their written informed consent to participate in this study. Written informed consent was obtained from the individual(s) for the publication of any potentially identifiable images or data included in this article.

## Author contributions

TXu: Writing – original draft. JinC: Writing – original draft. TXua: Data curation, Writing – review & editing. JiaC: Writing – review & editing. HL: Writing – review & editing.
